# Outcomes of a homestead food production program on nutritional knowledge, dietary diversity, food security and empowerment of rural women in Tehran province, Iran

**DOI:** 10.1186/s12889-024-17658-z

**Published:** 2024-01-08

**Authors:** Neda Ezzeddin, Naser Kalantari, Morteza Abdollahi, Parisa Amiri, Bahareh Amini, Farid Zayeri

**Affiliations:** 1 Department of Community Nutrition, Shahid Beheshti University of Medical Sciences, Tehran, Iran; 2 Faculty of Nutrition Sciences and Food Technology, and Social Determinants of Health Research Center, National Nutrition and Food Technology Research Institute, Shahid Beheshti University of Medical Sciences, Tehran, Iran; 3 Research Centre for Social Determinants of Health, Research Institute for Endocrine Sciences, Shahid Beheshti University of Medical Sciences, Tehran, Iran; 4 Proteomics Research Center, Department of Biostatistics, Shahid Beheshti University of Medical Sciences, Darband Street, Tehran, Iran

**Keywords:** Homestead food production, Dietary diversity, Nutritional knowledge, Women’s empowerment, Food security, Home garden, Structural equation modelling

## Abstract

**Background:**

Food and nutrition insecurity is a major health issue in developing countries. Homestead food production (HFP) programs are strategies for improving food and nutrition security of a country. Iran implemented a HFP program entitled “Nutrition Improvement of Rural and Nomadic Women” in its villages for a five-year period from 2017. The current study assessed the outcomes of this mentioned program and its determinants among rural women in Tehran province.

**Methods:**

The population of this cross-sectional study comprised a group covered by the program (*n* = 143) and a non-covered group (*n* = 160). The participants were selected randomly from 32 villages of five counties in Tehran province. Data was collected using five questionnaires: (1) General information, (2) Women’s empowerment, (3) Nutritional knowledge, (4) Dietary diversity, and (5) Household Food Insecurity Access Scale (HFIAS). Data was analyzed using the IBM SPSS version 21 and the IBM Amos SPSS version 22 software.

**Results:**

The results of the study showed no significant improvement in the expected indicators, such as frequency of home gardening, nutritional knowledge, dietary diversity, women’s empowerment, and household food insecurity status among women covered by the program (*p* > 0.05). The structural equation modelling (SEM) results indicated that women’s empowerment from the dimension of decision-making power (*r* = 0.151) and nutritional knowledge (*r* = 0.135) were the significant positive predictors of dietary diversity (*p* < 0.05), while household food insecurity predicted it negatively (*r*=-0.138) (*p* < 0.05).

**Conclusion:**

Because the current evaluated program has not been able to improve the expected indicators, modification of the program implementation may be needed, such as: addressing the probable barriers and challenges and strengthening the facilities in the covered villages. The current study presents a model of the dietary diversity predictors for consideration in redesigning or enhancing the program.

## Introduction

Food and nutrition insecurity is a major health concern in developing countries [1, 2]. Goal Two of the Sustainable Development Goals (SDG_s_), i.e., *“*End hunger, achieve food security and improved nutrition, and promote sustainable agriculture,” emphasizes improving the food security and nutrition situation [3]. According to studies and reports, rural areas are more vulnerable to food insecurity [2, 4]. In Iran, a systematic review indicated that rural inhabitants are more exposed to food insecurity (66.1%) compared to urban areas (47.1%) [2]. In Dehrashid et al. reported a high prevalence of food insecurity (80%) in villages in Iran [5]. Prevalence rates of 66%, 73%, and 69% in different rural areas of Zahedan [6], Bushehr [7], and in Kohkiluyeh and Boyer-Ahmad have also been reported respectively. One study showed that food security status deteriorated during the COVID-19 epidemic in rural areas of Iran [8]. Food insecurity has adverse consequences on both physical [9] and mental [10] health, specifically in rural areas [11].

The nutrition-sensitive agriculture (NSA) strategy has been widely adopted in order to improve the food and nutrition security status in villages [12–17]. NSA programs target the factors underlying under-nutrition in multiple dimensions [18]. Training and provision of facilities for the production of foods through home gardening, chicken and eggs, aquatics, etc., typical components of such programs [18], are associated with increased access to valuable food and money earning [19]. As the target group of these programs is usually the women, women’s empowerment and reduction in gender inequality are other achievements [20]. Based on the existing model, women’s empowerment is associated with improved nutritional status in the household [21]. The various types of NSA programs include: biofortification, homestead food production (HFP), and livestock transfer among others [22]. In HFP programs, women are trained to produce nutritious food in their home gardens or raise poultry near their homes [23]. An HFP program entitled “Nutrition Improvement of Rural and Nomadic Women” has been implemented in the villages of Iran since 2017 in an attempt to achieve Goal 2 of SDG_s_. This 5-year, inter-sectoral program was developed and implemented in cooperation with the Ministry of Health and Medical Education (Department of Community Nutrition) and the Ministry of Agriculture-Jihad (Office for Development of Agricultural Activities of Rural and Nomadic Women). The program focuses on training and establishing home gardens with vegetable production, healthy eating to improve access to micronutrient-rich foods, and promoting healthy eating patterns [24].

As the outcomes of this program have not been evaluated prior to the current study, this research purposed to investigate the expected results, including women’s nutritional knowledge, empowerment status (in two dimensions of “control over and access to financial resources” and “decision-making power,” household food security status, and women’s dietary diversity (DD) in rural areas of Tehran province. DD is recognized as a key element in high-quality diets and as a measure of dietary quality [25, 26], and it includes HFP programs [12, 13]. Therefore, the present study provided factors predicting DD as structural equation modeling (SEM) in addition to an evaluation of the outcomes of the program’s implementation. The results can be used by policymakers and planners of HFP programs.

## Methods

### Study design, population, and data gathering

This cross-sectional study was conducted among rural women in Tehran province counties from January to October 2022. Four out of eight counties (Ray, Islamshahr, Mallard, and Varamin) were purposefully selected from regions covered by the program because they continued with the program (From September 2018 to January 2022) and provided a larger number of covered villages. In total, 143 women were selected randomly (proportional to size) from 22 out of 25 considered villages that provided the information regarding women covered by the program (*n* = 458). The inclusion criteria was their interest in participating in the study, being less than 70 years old, and fully answering questions (at least 90%). The non-covered (*n* = 160) were selected randomly from 10 rural health centers or houses in five counties (Ray, Islamshahr, Mallard, Varamin, and Shahryar) and proportional to size. To prevent the spill-over of the program, these selected centers or village houses were located in the district where the program was not implemented. The inclusion criteria was being less than 70 years old, married or head of household, interested in participating in the study, and completing the questionnaires (at least 90%). It should be noted that the number of non-covered women was first 172 but was decreased during age matching between the two groups (*n* = 12).

### Measures

#### *Demographic and socio-economic information*

Demographic and socio-economic information was gathered by a questionnaire that include questions on participants’ age and marital status, the educational level and employment status of participants and head of households, the household monthly income, home area, and life conveniences (e.g., refrigerator, television, vacuum cleaner, washing machine, stove, telephone, cell phone, internet, laptop or computer, bicycle, car or motorcycle). This questionnaire also collected information on agricultural or livestock production (milk or eggs) and home gardening of the household.

#### *Dietary diversity (DD) assessment*

The guidelines proposed by the Food and Agriculture Organization of the United Nations (FAO) were observed [27] in assessing the individual DD of the studied women. The original version of this questionnaire could not be used, as it needed some modifications to fit the studied population. Therefore, it was translated and adapted based on the available food items and modified by a panel of Iranian nutritionists (*N* = 5). The questionnaire included questions about 16 food groups at the individual level: (1) cereals; (2) white roots and tubers; (3) vitamin A-rich vegetables and tubers; (4) dark leafy green vegetables; (5) other vegetables; (6) vitamin A-rich fruits; (7) other fruits; (8) organ meats; (9) flesh meats; (10) eggs; (11) fish and seafood; (12) legumes, nuts, and seeds; (13) milk and milk products; (14) oils and fats; (15) sweets, and (16) spices, condiments, and beverages. To measure DD, a 24-hour dietaryrecall was completed by each participant. Consumed (at least half of a serving) and unconsumed items in the dietary diversity questionnaire were scored 1 and zero points, respectively. Participants were asked again about unconsumed items, and they were scored zero if the answer was still negative. After merging groups 1 and 2 (starchy staples), 3 and 6 (other vitamin A-rich fruits and vegetables), 5 and 7 (other fruits and vegetables), and 9 and 11 (meat and fish), the questionnaires were scored. Scores ranged between 0 and 9, because the group of fats and sweets was not included.

#### *Household food insecurity access scale (HFIAS)*

In this study, a 9-item, 4-Likert scale (frequency-of-occurrence) questionnaire was used to assess the status of household food insecurity, during the previous month. This scale examined the respondents’ perceptions (for example, worrying about access to enough food) and behavioral responses (for example, consuming fewer or skipping meals) to food accessibility status of household members [28]. A literature review indicated that this scale is widely used to measure household food insecurity status [29–32]. In Iran, the validity and reliability of the Persian version of the questionnaire was confirmed by Mohammadi et al. (Cronbach’s alpha = 0.85) [33]. The questionnaire is scored based on the frequency of food insecurity occurrence (most of the time = 3; sometimes = 2; rarely = 1; and never = 0), and possible scores range from 0 to 27. A higher score indicates a more severe household food insecurity status [34].

#### *Rural women’s empowerment assessment*

The current study examined two dimensions of rural women’s empowerment status, i.e., (“control over and access to financial resources” and “decision-making power,”) using the questionnaire developed and validated by Savari et al. [35]. The section regarding “control over and access to financial resources” included questions about women’s ownership of resources (income, savings, livestock, agricultural land, etc.), and their degree of control over these resources toward household food security. For example, women were asked, “To what extent are you allowed to take livestock products for household use?” or “Are you cultivating a product that will generate income for yourself?” or “To what extent are you allowed to use household income?” This section contained 13 questions scored on a Likert scale (from never = 1 to very much = 5). The “decision-making power” section included 7 questions that examine the power of women in making decisions toward food security in the household and was scored like the previous section. Questions included, for example, “To what extent is your opinion considered in the purchase of food for household consumption?” or “To what extent is your opinion considered in the consumption of products in the household?” Because it was not common for everyone to have household products (either agriculture or livestock), only women who met that particular criteria answered such questions. The mean score of every section was considered in statistical analysis. Cronbach’s alpha was 0.77 for control over and access to financial resources and 0.75 for decision-making power.

#### *Nutritional knowledge assessment*

In this study, the nutritional knowledge questionnaire was used to assess the status of nutritional knowledge among the participants. This questionnaire was validated by Heshmat in a national study with an acceptable Cronbach’s alpha of 0.79 [36, 37]. Areas assessed in the current studycomprised the reason for eating food; identification and role of food groups; sources of protein other than meat, and sources of micronutrients. These areas were evaluated through 18 questions which were scored 1 to 6 (for a possible total of 52 points). One point was given to each correct answer mentioned by the participants. The reliability (Cronbach’s alpha) of the questionnaire was 0.88 in the current study.

### Statistical analysis

The qualitative variables were described using frequency distribution tables, and the quantitative variables were described using statistical indices like mean and standard deviation. To compare the quantitative variables between the two studied groups, the independent samples T-test and simple linear regression model were applied. The chi-square test was also used to compare the qualitative variables between the two groups. The women’s empowerment dimension of control over and access to financial resources was compared between the two groups using the multiple linear regression model. The simple linear regression model was also applied to assess the DD determents. Finally, the SEM approach was utilized to identify the impact pathways of variables among the entire studied population. Statistical analysis was performed using IBM SPSS and IBM Amos version 22.0. A *p*-value less than 0.05 was considered as statistically significant.

## Results

Most of the participants in both groups were housekeepers with a primary and secondary education. Tables 1 and 2 present the characteristics of the studied population. To reduce the number of socio-economic status (SES) variables, principal component analysis (PCA) was applied to construct an SES variable. The studied population had the necessary criteria in terms of sampling adequacy and correlation between variables (KMO = 0.7; Bartlett test *p*-value < 0.001). Based on the results, the main factor with the highest described variance (highest eigenvalue) was selected as the SES variable. This factor consisted of the educational level of the woman and the head of household, occupational status of the household head, and the monthly household income. The simple regression test showed no significant difference in the variables of SES and participants’ age in both groups (*p* > 0.05).

**Table 1:** The characteristics of the study population (quantitative variables)

Quantitative variables	Covered Group N = 143	Non- Covered Group N = 160
Age (year)	43.43 ± 9.30^a^	42.10 ± 8.54
*Household size*	3.86 ± 1.23	3.74 ± 1.10
*Household monthly income (Million Tomans)*	5.64 ± 3.81	5.72 ± 3.35
Life facilities *(score)*	8.1 ± 1.8	8.5 ± 1.6

^a^Mean± Standard Deviation

**Table 2:** The characteristics of the study population (qualitative variables)

Qualitative variables	Covered Group N = 143	Non- Covered Group N = 160
Marital status		
*Married* *Single/Widowed/divorced*	129 (90.2)^a^ 14 (9.8)	148 (92.5) 12 (7.5)
Educational level of woman		
*Illiterate* *Primary and secondary school* *High school and diploma* *Associate Degree and Bachelor* *Masters and higher*	12 (8.4) 73 (51/0) 47 (32.9) 11 (7.7) 0	16 (10.0) 73 (45.6) 55 (34.4) 16 (10.0) 0
Educational level of head of household		
*Illiterate* *Primary and secondary school* *High school and diploma* *Associate Degree and Bachelor* *Masters and higher*	15 (10.5) 85 (59.4) 33 (23.1) 9 (6.3) 1 (0.7)	11 (6.9) 90 (56.3) 47 (29.4) 11 (6.9) 1 (0.6)
Occupational category of participants		
*Unemployed/Housekeeper* *Worker/ farmer/ rancher* *Self-employed /shopkeeper/driver* *Employee/Teacher/Military* *Retired*	122 (85.3) 7 (4.9) 4 (2.8) 10 (7/0) 0	145 (90.6) 5 (3.1) 3 (1.9) 6 (3.8) 1 (0.6)
Occupational category of household head		
*Unemployed/Housekeeper* *Worker/ farmer/ rancher* *Self-employed /shopkeeper/driver* *Employee/Teacher/Military* *Retired*	19 (13.3) 14 (9.8) 65 (45.5) 35 (24.5) 10 (7.0)	16 (10.0) 17 (10.6) 50 (31.3) 62 (38.8) 15 (9.4)
The status of home garden with vegetable production		
*Yes*	20 (14.0)	17 (10.6)
The status of agricultural production		
*Yes*	31 (21.7)	6 (3.8)
The status of animal production		
*Yes*	23 (16.1)	8 (5.0)
The status of membership in village associations		
*Yes*	55 (38.5)	27 (16.9)

^a^Number (Percent)

The frequency of home gardening in the two groups was compared by chi-square test. The results indicated that the number (%) of active home gardens in the covered and non-covered groups was 20 (14.0%) and 17 (10.6%), respectively, which was not significantly different (*p* = 0.372). It is noteworthy that three of the twenty home gardens were established because of the HFP program.

The expected outcomes of the program, i.e., nutritional knowledge, women’s empowerment status from the dimensions of “decision-making power” and “control over and access to financial resources,” household food security status, and DD, were compared between groups using the independent samples T-test. The results revealed no significant difference between the two groups regarding the investigated variables (Table 3), except for the mean score of “control over and access to financial resources”. However, the difference was not significant when the association was reexamined after adjusting for other variables, including status of agricultural, animal and home garden production, and the status of membership in village associations, in multiple linear regression (*p* > 0.05).

**Table 3:** The difference between the covered and non-covered groups

Variables	Covered Groups N = 143	Non- Covered Groups N = 160	P.value
Dietary diversity *(score)*	4.74 ± 1.26^a^	4.91 ± 1.22	0.233
*Household food insecurity (score)*	5.79 ± 6.21	5.52 ± 5.77	0.693
Women’s decision-making power *(mean score)*	3.73 ± 0.84	3.60 ± 0.97	0.221
Women’s control over and access to financial resources *(mean score)*	1.57 ± 0.47	1.47 ± 0.38	0.042
Nutritional Knowledge *(score)*	17.20 ± 6.89	16.70 ± 8.82	0.584

Note: *p*-value less than 0.05 was deemed to be statistically significant

To identify the determinants of DD, both groups were analyzed as a community because of the relative homogeneity (*N* = 303). Table 4 shows the results of the association between DD and the studied variables in the simple linear regression model. Based on the results, factors including: household SES (B = 0.216, *p* = 0.003), household food insecurity (B=-0.035, *p* = 0.004), women’s empowerment from the dimensions of “decision-making power” (B = 0.199, *p* = 0.011) and “control over and access to financial resources” (B = 0.359, *p* = 0.031), nutritional knowledge (B = 0.024, *p* = 0.008) and women’s membership in village associations (B = 0.348, *p* = 0.031), were identified as predictors of DD (*p* < 0.05).

**Table 4:** Simple linear regression to examine the association of dietary diversity and studied variables among rural women

Variables	B	S.E^a^	P.value
Participants’ age	0.003	0.008	0.694
SES^b^	0.216	0.071	0.003
*Household food insecurity*	−0.035	0.012	0.004
Women’s control over and access to financial resources	0.359	0.166	0.031
Women’s decision-making power	0.199	0.077	0.011
Nutritional Knowledge	0.024	0.009	0.008
The status of home garden with vegetable production in (Yes = 1, No = 0)	0.130	0.219	0.552
The status of agricultural production (Yes = 1, No = 0)	0.161	0.219	0.462
The status of animal production (Yes = 1, No = 0)	−0.244	0.236	0.303
The status of membership in village associations (Yes = 1, No = 0)	0.348	0.160	0.031

^a^Standard Error

^b^Socio-Economic Status

**Fig. 1:**
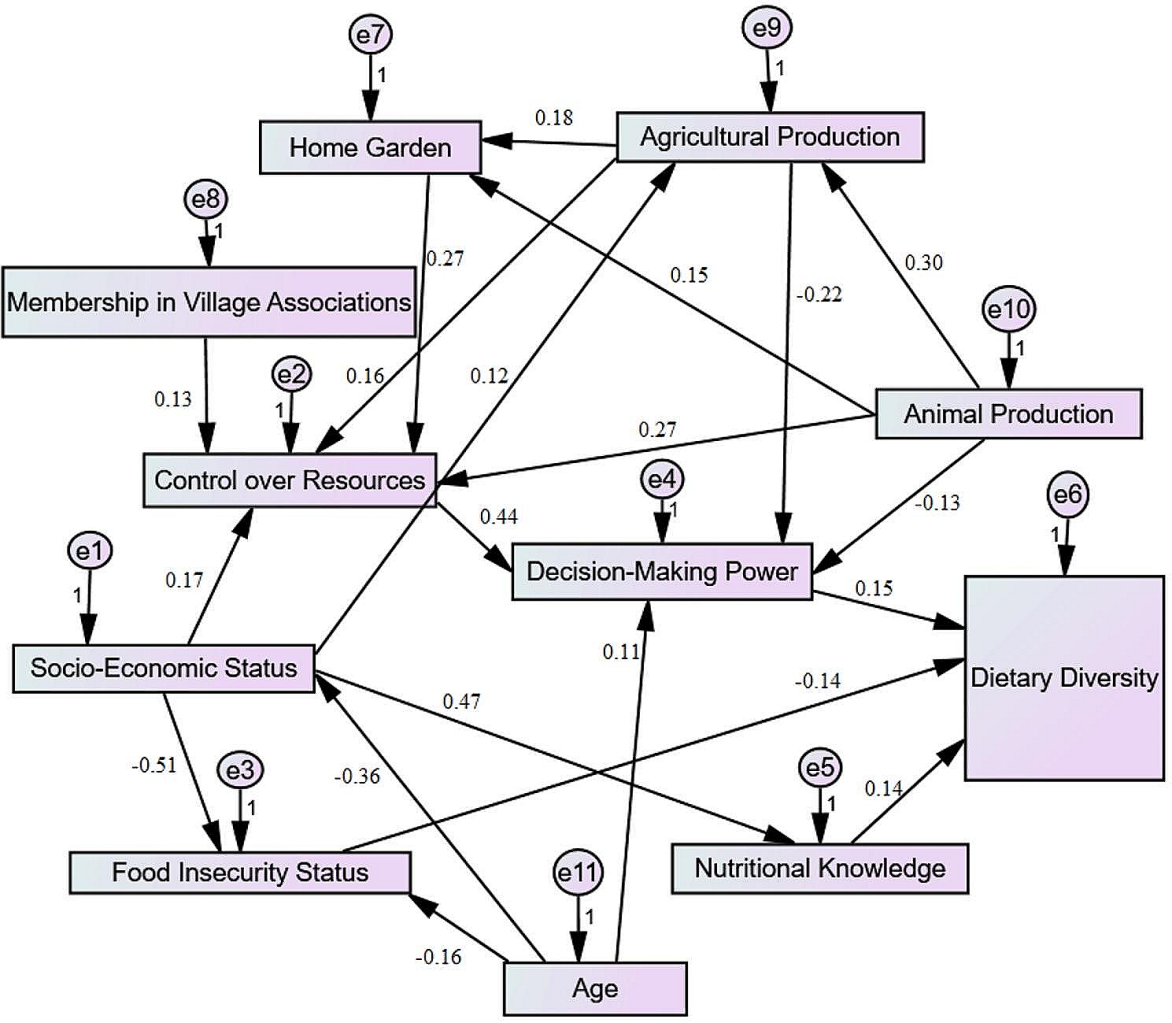
Proposed model for predicting the dietary diversity predictors among rural women

SEM methodology was then applied to show the impact pathways of the studied variables (Fig. 1; Table 5). All pathways presented in the model were significant (*p* < 0.05); additional pathways were removed to improve the fit indices. In the proposed model, women’s nutritional knowledge (*r* = 0.14, *p* = 0.017) and decision-making power (*r* = 0.15, *p* = 0.007) were positive predictors of DD, while the food insecurity score was negative (*r*=-0.14, *p* = 015). SES did not have a direct impact on DD, but it was indirectly associated through other variables (household food security, nutritional awareness, etc.). Although the women’s control over and access to financial resources was not directly associated with DD, it was a predictor of women’s decision-making power (*r* = 0.44, *p* < 0.001). The status of agricultural (*r* = 0.16, *p* = 0.002), animal (0.27, *p* < 0.001), or home garden (0.27, *p* < 0.001) production was also a predictor of women’s control over and access to financial resources.

**Table 5:** Summary of model for predicting the dietary diversity determinants among rural women

Proposed model	Estimate	S.E.	C.R.	P-value
Pathways				
Dietary Diversity <--- *Food Insecurity Status*	−0.029	0.012	−2.423	0.015
Dietary Diversity <--- Decision-making power	0.205	0.76	2.71	0.007
Dietary Diversity <--- Nutritional Knowledge	0.021	0.009	2.379	0.017
***Fit indices***	**CMIN/DF**: 0.970		**TLI**: 1.004	
	**GFI**: 0.980		**IFI**: 1.002	
	**CFI**: 1.000		**RMSEA**: <0.001	

CMIN/DF = minimum discrepancy function by degrees of freedom divided; TLI = Tucker-Lewis index; GFI = goodness of fit index; IFI = Incremental Fit *Index;* CFI = comparable fit index; RMSEA = Root mean square error of approximation

## Discussion

The present study assessed the outcomes of an HFP program in women living in the rural villages of Tehran province. The results did not show a significant difference in the expected indicators, i.e., frequency of home garden with vegetable production, nutritional knowledge, DD, women’s empowerment, and household food security status, between the two groups (covered and non-covered groups in the program). Subsequent analyses were performed to determine the predictors of DD in all the studied population of both groups. The results indicated that women’s empowerment from the dimension of decision-making power and nutritional knowledge predicted DD positively, while household food insecurity predicted it negatively.

A diverse diet is the basis of a healthy diet that provides the required calories, fat, protein, and micronutrients [14]. Based on the current results, no significant difference in DD scores was observed between the covered and non-covered groups. According to a study conducted by Olney et al. in Cambodia, an HFP program was associated with better DD status in the intervention households [12]. In another study by Talukder et al. conducted in Bangladesh, Cambodia, Nepal, and the Philippines, DD was significantly increased by implementing a HFP program [13]. Such an association was also seen in a systematic review conducted by Margolies et al. [15]. Nevertheless, some studies have obtained results consistent with the results of the current study. For example, Rosenberg evaluated the outcomes of a four-year implementation of an HFP program in Zambia and found no significant difference in DD between the studied groups [14]. In a study conducted by Rahman et al. in Bangladesh, the food intakes were not satisfactory despite the increase in home garden production. The preference of households to earn money by selling products to meet other household needs and the lack of a previous habit of consuming vegetables explained this finding [16]. Kumar also found no significant improvement in household DD, despite increases in the amount and variety of products. The researchers believed that increasing the variety of production or improving women’s empowerment is not enough to improve the diet in a household. Attention to other areas such as increasing awareness, information, and linking with markets is needed to achieve improvement in action [17]. In the current study, the program implementation was not associated with a significant increase in the number of established home gardens, nutritional knowledge, women’s empowerment, etc. Therefore, the paths to improving DD have not been fulfilled.

Women’s empowerment is one of the objectives of HFP programs, including the currently evaluated program. Women’s empowerment is associated with a more sustainable and equitable food system, which is accompanied by better nutrition and food security status for all [38]. The lack of difference in women’s empowerment indicators in the present study can be explained by the absence of a significant difference in the number of home gardens between the two groups, because it is expected that women’s control over resources and their agency will increase through earning money from the sale of extra products [19]. As seen in the current study, having a home garden with vegetable production is associated with increased women’s control over and access to financial resources. The model proposed in the current study indicated that women’s control over and access to financial resources is positively associated with decision-making power in women. It seems that women’s control over resources increases the bargaining power, which leads to more women’s agency and decision-making power in allocating resources in the household [39, 40]. It was also shown that greater decision-making power in women is associated with a higher DD score. In the study conducted by Merga et al. in Ethiopia on women of reproductive age, women’s empowerment on purchasing foods increased DD about four-fold [41]. In other study by Gudeta in Ethiopia, the more women’s empowerment, which also contained the dimension of decision-making power, increased DD three-fold [42]. The same result was also seen in Nigeria by Voufo et al., where the increase in women’s empowerment was positively associated with DD, and it was stronger in households in which the share of women’s decision-making was higher [43]. The extent to which women have access to and control over resources largely determines the status of the care provided for their children and other household members. The results of a study in Ethiopia showed that all indicators of women’s empowerment were positively associated with better DD for children and women [44]. Evidence from Bangladesh also showed that women’s participation in household decision-making and the ability to purchase food (an aspect of empowerment) are significantly related to the availability of a varied diet in the household [45]. Nonetheless, a few studies are inconsistent with the current one. In the study conducted by Harris-Fry in rural Bangladesh, women’s involvement in decision-making was not a predictor of DD or food security [46]. The reporting of this relationship by the majority of existing studies reinforces the idea that women’s decision-making power is an important aspect of women’s empowerment, as it leads to women’s DD and thus better nutritional status [47].

Regarding the proposed model, the food insecurity score was inversely associated with SES. Household SES including educational level and household income was also recognized as a predictor of food insecurity in the rural areas of Iran [5, 48–50]. In this study, SES did not impact DD directly but through the impact on food insecurity. It has been shown that food insecurity is associated with the low quality of a diet [51], and DD was recognized as a suitable indicator for the probability of nutrient adequacy in women from Tehran by Tavakoli et al. [52]. The results of their study showed that food insecurity was negatively associated with DD; as the food insecurity score increased, the DD score decreased. Gudeta et al. also reported that DD was associated with food security among the studied women [42]. This association was also shown in other studies, such as Hosseinpour in Tehran, Iran [53]; Sheikhi in Zahedan, Iran [6], in Karachi, Pakistan [54]; and Binte Ali in rural Bangladesh [55]. The importance of food security in achieving DD has led to the use of DD as a proxy indicator of food security [56]. Therefore, to benefit from DD in society, food insecurity must be eliminated by addressing its underlying factors.

Healthy nutrition training is presented to rural women as an integral part of the NSA programs [13, 19]. Studies indicate a lower level of nutritional knowledge in rural women compared to urban ones [57, 58]. The results of the current study did not show a significant difference in the nutritional knowledge scores of covered and non-covered groups in the program. Jones et al., however, evaluated a program similar to the one in the current study and observed a significant increase in the level of nutritional knowledge in the intervention group compared to the control 36 months after the implementation of the program [59]. In another study in Burkina Faso, women participating in a NSA program still had better nutritional knowledge than the control group four years after their participation [60]. Considering the lack of significant change in nutritional knowledge among the women covered in the program, it is necessary for planners and executors to assess the status of training reach as well as its quality, quantity, and continuity. In this study, SES was also identified as a positive predictor of nutritional knowledge. This finding is consistent with the studies of Heshmat et al. [37] and Salehi et al. [58] in Iran. In the study conducted by Vriendt among Belgian women, the roles of education and employment status were recognized in determining nutritional knowledge status [61]. Because SES was a predictor of nutritional knowledge in the proposed model, and because about half of the women participating in the study had low educational level (primary and secondary school), the expansion of quality nutrition training in rural areas can improve the nutritional knowledge of rural women. On the other hand, nutritional knowledge in women had a positive association with their DD. In the study conducted by Melesse in urban Ethiopia, nutrition knowledge was positively associated with more DD [62]. It has been shown that nutrition training among pregnant women in Malawi increased DD by improving their nutrition perceptions and behaviors [63]. However, in the study conducted by Agyei et al. on women from Northern Ghana, nutritional knowledge was not associated with DD, and income played a more important role in determining DD status. It seems that nutritional knowledge should be considered as a determent factor in accompaniment with other predictors of DD, not individually.

The current study is one of the few studies that has evaluated an HFP program among an Iranian population. It also provides predictors of DD as a model by examining diverse variables. Due to the lack of information about the status of the examined indicators before the intervention of the program, it was not possible to compare them. Although the two groups were matched in terms of age and SES, longitudinal evaluation assessments can provide a more accurate picture of the impact of the program. Finally, part of the population of the counties of Tehran province comprises non-natives and immigrants who are economically poor, and this fact can affect the results of program implementation. Therefore, it is recommended that future studies evaluate the results of the program implementation in other provinces as well.

## Conclusions

The current study was conducted among rural women of Tehran province, Iran, to assess the expected outcomes of an HFP program. Based on the results, no significant differences exist in the studied variables between the group covered by the program and the non-covered group. It was also seen that having a home garden can improve DD indirectly, and nutritional knowledge affects it directly. This result indicates that if program implementation is accompanied by an increased number of established home gardens and more nutritional knowledge, it can improve the DD of the participants. Because the currently evaluated program has not been able to improve the expected indicators, modifying it may be necessary, as in addressing the probable barriers and challenges and strengthening the life conveniences in the villages.

## Data Availability

According to the research project contract, the researchers are not allowed to share the data directly, but the data will be available through correspondence with the Vice-Chancellor of Research Affairs (Mpajouhesh@sbmu.ac.ir).
